# A Multifactorial Azole Response in Clinically Relevant *Fusarium* Species Complexes

**DOI:** 10.1111/myc.70220

**Published:** 2026-09-05

**Authors:** Patrícia Helena Grizante Barião, Ludmilla Tonani, Adriana Rodrigues Alves, Otávio Guilherme Gonçalves de Almeida, Cledir Santos, Marcia Regina von Zeska Kress

**Affiliations:** ^1^ Department of Clinical Analyses, Toxicology, and Food Science, School of Pharmaceutical Sciences of Ribeirao Preto University of Sao Paulo Ribeirao Preto SP Brazil; ^2^ Scientific and Technological Bioresource Nucleus (BIORENUFRO) Universidad de La Frontera Temuco Chile; ^3^ Postgraduate Program in Biotechnology Federal University of Technology‐Paraná Ponta Grossa PR Brazil

**Keywords:** antifungal resistance, azoles, *CYP51A*, efflux pumps, *Fusarium* spp., intrinsic resistance, voriconazole

## Abstract

**Background:**

*Fusarium* spp. are clinically relevant filamentous fungi with intrinsically low susceptibility to multiple antifungal agents, particularly azoles, and are included in the World Health Organization fungal priority pathogens list.

**Objectives:**

To investigate azole resistance‐related mechanisms in clinical isolates belonging to the *Fusarium solani* species complex (FSSC), *Fusarium oxysporum* species complex (FOSC) and *Fusarium fujikuroi* species complex (FFSC) recovered in São Paulo, Brazil.

**Methods:**

Twenty‐six clinical isolates were identified by sequencing of the ITS region, *TEF1* and *RPB2*. Antifungal susceptibility testing, *CYP51A* amplification and sequencing and expression analysis of *CYP51A* and *ABC1* under voriconazole exposure were performed.

**Results:**

Antifungal susceptibility testing showed broadly elevated azole MICs, with the FSSC displaying the least favourable profile. Comparative CYP51A analysis revealed distinct amino acid signatures among the three species complexes, including residues at positions equivalent to canonical azole resistance hotspots in *Aspergillus fumigatus* . However, no simple relationship was observed between CYP51A amino acid profiles and azole MIC values. In contrast, voriconazole exposure induced marked *CYP51A* upregulation in all tested isolates, with substantially higher expression in the higher‐MIC FSSC isolate. *ABC1* was also upregulated in FSSC isolates, although at markedly lower levels than *CYP51A*.

**Conclusions:**

These findings support a multifactorial model of azole response in *Fusarium* species complex strains, involving conserved CYP51A sequence features, strong target‐gene induction under antifungal stress, and a lower but detectable contribution of efflux‐associated mechanisms.

## 1 Introduction

Species of the genus *Fusarium* are ubiquitous filamentous fungi widely distributed in soil, water, air and plant‐associated environments. Although traditionally recognised as important phytopathogens, they are now also established as opportunistic agents of animal and human disease, reflecting broad ecological versatility and a remarkable capacity for adaptation across distinct biological niches [1]. In humans, *Fusarium* spp. are associated with a wide spectrum of clinical manifestations, including keratitis, onychomycosis, localised infections and disseminated fusariosis, particularly in severely immunocompromised patients [2, 3]. Among the species complexes most frequently associated with human infection, the *Fusarium solani* species complex (FSSC), *Fusarium oxysporum* species complex (FOSC) and *Fusarium fujikuroi* species complex (FFSC) are especially relevant, with members of the FSSC accounting for a substantial proportion of reported fusariosis cases [3, 4]. Although taxonomic revisions have proposed the transfer of FSSC members to the genus *Neocosmospora*, the term *Fusarium* remains widely used in medical mycology and clinical microbiology because nomenclatural stability is considered important for communication, diagnosis and patient management [2, 5]. Accordingly, this terminology is retained throughout the present study.

The clinical management of fusariosis remains challenging because *Fusarium* species frequently display intrinsically low susceptibility to multiple antifungal agents, including azoles, polyenes and echinocandins [3, 6, 7]. This clinical relevance has been further underscored by the inclusion of *Fusarium* spp. in the World Health Organization fungal priority pathogens list within the high‐priority group, reflecting their impact on human health, intrinsic antifungal resistance, limited therapeutic options, and the need to strengthen laboratory capacity, surveillance and research efforts [8, 9]. Among the available therapeutic options, triazoles, particularly voriconazole, remain clinically relevant and are often recommended as first‐line agents, alone or in combination with liposomal amphotericin B; however, treatment efficacy is frequently limited by the complexity of the underlying resistance mechanisms [10]. Voriconazole susceptibility profiles may vary substantially among species complexes and among isolates, reinforcing the need for accurate taxonomic identification and a better understanding of the molecular basis of azole response [3, 4]. At the same time, the relationship between reduced in vitro susceptibility and clinical response to therapy in fusariosis remains incompletely defined. This reflects not only the absence of clinical breakpoints for *Fusarium* spp. but also the complexity of these infections and their management, which often rely on limited clinical evidence and are influenced by multiple host‐ and treatment‐related factors [10, 11]. Azoles exert their antifungal activity by inhibiting lanosterol 14α‐demethylase, a key enzyme of the ergosterol biosynthesis pathway encoded by genes of the CYP51 family [12, 13, 14]. In *Fusarium*, this pathway is of particular interest because these fungi may harbour three *CYP51* paralogs (*CYP51A*, *CYP51B* and *CYP51C*), and previous studies have suggested that sequence variation in these genes may contribute to the intrinsically low susceptibility of this genus to azoles [3].

Despite these advances, the molecular basis of azole resistance in *Fusarium* remains incompletely understood. Azole resistance in fungi is increasingly recognised as a multifactorial phenotype that may involve target‐site modification, gene overexpression, upregulation of multidrug transporters, including efflux pumps and permeability barriers, and broader adaptive responses to antifungal stress [15]. In *Fusarium*, this broader framework is particularly relevant because structural variation in *CYP51* alone may not fully explain the resistant phenotype. Previous studies have shown that exposure to azole antifungals may induce overexpression of *CYP51A* in members of the FSSC, providing evidence that transcriptional regulation of the target gene may contribute to reduced susceptibility [16]. Likewise, efflux‐mediated mechanisms appear to contribute to the azole response of clinically relevant *Fusarium* species, as illustrated by the reported involvement of the ATP‐binding cassette (ABC) efflux pump *ABC1* in the innate azole resistance phenotype of *Fusarium keratoplasticum* and by its marked induction following voriconazole exposure [17]. Taken together, these observations support a broader view of azole resistance in *Fusarium* as a multifactorial process and provide the rationale for integrated approaches combining taxonomic identification, antifungal susceptibility testing, sequence analysis and transcriptional profiling. Accordingly, this study investigated isolates belonging to the FSSC, FOSC and FFSC by integrating multilocus molecular identification, antifungal susceptibility testing, *CYP51A* sequence analysis, and expression analysis of *CYP51A* and the efflux‐associated gene *ABC1* under voriconazole exposure.

## 2 Material and Methods

### 2.1 *Fusarium* spp. Clinical Isolates and Strains

A total of 26 clinical isolates of *Fusarium* spp. and two reference strains, *Fusarium keratoplasticum* ATCC 36031 and *Fusarium oxysporum* ATCC 48112, were included in this study. The clinical isolate set included representatives of the *Fusarium solani* species complex (FSSC; *n* = 17), *Fusarium oxysporum* species complex (FOSC; *n* = 6) and *Fusarium fujikuroi* species complex (FFSC; *n* = 3). The clinical isolates were obtained from different human biological specimens collected in Brazil (Table S1).

### 2.2 Ethics Statement

The authors confirm that the ethical policies of the journal, as noted in the author guidelines, have been adhered to. This study was approved by the Research Ethics Committee of the School of Pharmaceutical Sciences of Ribeirao Preto, University of Sao Paulo (FCFRP‐USP), under protocol no. 561/2021.

### 2.3 Artificial Intelligence Disclosure

During manuscript preparation, the authors used ChatGPT (OpenAI) for editorial support, including language revision, sentence restructuring and improvement of readability. All scientific content, data interpretation, conclusions and final decisions regarding the manuscript were reviewed and approved by the authors. No artificial intelligence tool was used to generate, analyse or interpret the study data, and no such tool is listed as an author.

### 2.4 DNA Extraction, PCR Amplification, Sequencing and Phylogenetic Analysis

Genomic DNA from *Fusarium* isolates was extracted from fungal mycelia according to the method described by Junghans and Metzlaff [18]. Molecular identification was performed by PCR using GoTaq Hot Start DNA polymerase (Promega, USA) and primer sets targeting the internal transcribed spacer (ITS) region of ribosomal DNA, the second largest subunit of RNA polymerase II (*RPB2*), and the translation elongation factor 1‐alpha (*TEF1*). The primers used were ITS1 (5′‐TCCGTAGGTGAACCTGCGG‐3′) and ITS4 (5′‐TCCTCCGCTTATTGATATGC‐3′) for the ITS region [19]; RPB2 forward (5′‐GGGGWGAYCAGAAGAAGGC‐3′) and RPB2 reverse (5′‐CCCATRGCTTGYTTRCCCAT‐3′) for *RPB2* [20]; and TEF1‐F (5′‐ATGGGTAAGGARGACAAAGAC‐3′) and TEF1‐R (5′‐GGARGTACCAGTSATCATGTT‐3′) for *TEF1* [21]. Following PCR amplification, the products were purified and sequenced using the same primers on an ABI 3100 Genetic Analyzer (Applied Biosystems, Foster City, CA, USA). Electropherograms were analysed using ChromasPro version 2.1.10 (Technelysium Pty Ltd). The resulting sequences were compared with reference sequences deposited in GenBank database of the National Center for Biotechnology Information (NCBI) using the Basic Local Alignment Search Tool (BLAST) for nucleotide [22, 23].

Phylogenetic analyses were performed in MEGA12 [24] using the Maximum Likelihood method under the Kimura 2‐parameter model [25]. Rate heterogeneity among sites was modelled using a discrete Gamma distribution with five categories together with a proportion of invariant sites. Initial trees for heuristic search were generated by the Neighbour‐Joining method [26], and node support was assessed by bootstrap analysis with 1000 replicates [27]. The same analytical framework was used for two concatenated datasets: *TEF1* + *RPB2* to resolve the major *Fusarium* species complexes, and ITS + *TEF1* + *RPB2* to improve phylogenetic resolution within the FSSC.

### 2.5 Antifungal Susceptibility Testing

Antifungal susceptibility testing was performed using the broth microdilution method according to the Clinical and Laboratory Standards Institute (CLSI) document M38‐A2 [28]. Amphotericin B (AMB), voriconazole (VOR), itraconazole (ITR) and posaconazole (POS) (Sigma, Aldrich–St. Louis USA) were tested over a concentration range of 0.06–32 μg/mL. Briefly, fungal isolates were cultured on Potato Dextrose Agar (PDA) at 28°C for 5 days. Conidial suspensions were prepared and adjusted in autoclaved distilled water to a final concentration of 0.4 × 10^4^ to 5 × 10^4^ conidia/mL. The inocula were then transferred to RPMI 1640 medium buffered with 0.165 M 3‐(N‐morpholino) propanesulfonic acid (MOPS), pH 7.0 and incubated at 35°C for 48 h. Minimum inhibitory concentrations (MICs) were determined by visual reading, and *Aspergillus flavus* ATCC 204304 was used as the quality control strain [28]. MIC range, MIC50, MIC90 and geometric mean (GM) values were calculated using Microsoft Excel 2019 (version19.08). MIC50 and MIC90 were defined as the lowest antifungal concentrations able to inhibit 50% and 90% of the isolates, respectively. Statistical analyzes were performed using a one‐way test, adopting a significance level of 5% (*p* < 0.05). Because clinical breakpoints have not been established for *Fusarium* species, epidemiological cutoff values (ECV) were used for interpretation of wild‐type (WT) and non‐wild‐type (NWT) with respect to antifungal susceptibility [11]. For the FSSC, the 97.5% ECVs were 8 μg/mL for amphotericin B and 32 μg/mL for itraconazole, posaconazole and voriconazole; for the FOSC, the corresponding ECVs were 8 μg/mL for amphotericin B and posaconazole, 16 μg/mL for voriconazole and 32 μg/mL for itraconazole. All experiments were performed with two biological replicates and technical triplicates.

### 2.6 *CYP51A* Primer Design

As no suitable primers were available for amplification of *CYP51A* in isolates belonging to the FSSC, FOSC and FFSC, primers were designed in this study using two complementary sources of sequence information. First, representative public genome assemblies of *Fusarium* species were retrieved from NCBI, selected according to assembly quality assessed with QUAST v.5.0.2 [29], and subjected to gene prediction with AUGUSTUS v.3.4.0 [30]. Candidate *CYP51* coding sequences were identified by BLASTx searches against a custom database built from *Fusarium* CYP51 proteins retrieved from UniProtKB using DIAMOND v.0.9.14.115 [31]. Second, annotated *CYP51A* nucleotide sequences from representative species of the FSSC, FOSC and FFSC were retrieved from GenBank and used to complement the alignments. Multiple sequence alignment was performed with Clustal Omega v.1.2.4 [32], and phylogenetic analysis was carried out in MEGA X with the Neighbour‐Joining method and 1000 bootstrap replicates [33]. Conserved regions identified from these analyses were used for primer design.

### 2.7 *CYP51A* Amplification, Sequencing and Sequence Analysis

The *CYP51A* gene was amplified using the primer sets designed in this study and TransStart FastPfu High‐Fidelity DNA Polymerase (TransGen/Synapse). PCR products were purified and sequenced using the same primer on an ABI 3100 Genetic Analyzer (Applied Biosystems, Foster City, CA, USA). Sequence chromatograms were inspected and edited in ChromasPro v.2.1.10 (Technelysium Pty Ltd). The resulting CYP51A coding sequences were aligned and compared using Clustal Omega and MEGA v.11.0.11 to identify nucleotide variation among the clinical isolates.

In addition, a phylogenetic analysis of CYP51A coding sequences was performed in MEGA12 [24] using the Maximum Likelihood method under the Kimura 2‐parameter model [25]. Rate heterogeneity among sites was modelled using a discrete Gamma distribution with five categories together with a proportion of invariant sites. Node support was assessed by bootstrap analysis with 1000 replicates [27]. Initial trees for heuristic search were generated using the Neighbour‐Joining method [26].

### 2.8 Antifungal Exposure for Gene Expression Analysis

Three clinical isolates and two reference strains were selected for gene expression analysis in order to represent distinct voriconazole susceptibility profiles within the *Fusarium solani* species complex (FSSC) and the *Fusarium oxysporum* species complex (FOSC). In the FSSC, *F. keratoplasticum* LMC7108.01 (VOR MIC = 8 μg/mL) and LMC7113.02 (VOR MIC > 32 μg/mL), together with the reference strain ATCC 36031 (VOR MIC = 16 μg/mL), were included. In the FOSC, *F. oxysporum* LMC7137.01 (VOR MIC = 4 μg/mL), together with the reference strain ATCC 48112 (VOR MIC = 16 μg/mL), was included. Antifungal exposure was performed with modifications of the protocol described by James et al. [34]. Conidia from *Fusarium* spp. clinical isolates and reference strains were produced on Potato Dextrose Agar (PDA) at 30°C for 6 days. Conidial suspensions were prepared in sterile distilled water and adjusted to a final concentration of 1 × 10^4^ conidia/mL. Each suspension was inoculated into 10 mL of Potato Dextrose Broth (PDB) and incubated at 30°C, 150 rpm for 48 h. For antifungal induction, cultures were exposed to voriconazole (VOR) at 8 μg/mL VOR and incubated at 30°C, 150 rpm for 6 h. In parallel, control cultures without VOR were maintained under the same conditions. After incubation, mycelia were collected by filtration through filter paper (J. Prolab) and immediately frozen in liquid nitrogen for subsequent RNA extraction.

### 2.9 RNA Extraction, cDNA Synthesis and qPCR Analysis of *CYP51A* and *ABC*1 Expression

Total RNA was extracted from freeze‐dried mycelia biomass using the RNA Easy Fast Plant Tissue Kit (Tiangen, China), according to the manufacturer's instructions. RNA samples were treated with RQ1 RNase‐Free DNase (Promega), and first‐strand cDNA was synthesised using the Reverse Transcription System kit (Promega—A5001) with oligo dT primers, following the manufacturer's recommendations.

Gene expression analysis was performed for *CYP51A* and *ABC1*. For FSSC isolates, *CYP51A* amplification was carried out using primers NkCYP51A‐983‐F (5′‐CCTGGATCATGTTCCGTCTT‐3′) [34] and FSSCCYP51A‐R (5′‐GAATGTTGAAGAGGTGGAAGA‐3′) (this study). For FOSC isolates, *CYP51A* amplification was performed using the primers FOSCCYP51A‐F (5′‐CTTCTCATGGCTGGACAACA‐3′) and FOSCCYP51A‐R (5′‐GAGTTGCTCTTGGTAGAGTTCC‐3′) (this study). Expression of *ABC1* was assessed using primers FkABC1‐F3561 (5′‐CCTTCTGGGAGCAGCTTAAA‐3′) and FkABC1‐R3631 (5′‐TGAAGAGAGCAACCGAAATACA‐3′) (this study). *ABC1* expression was not evaluated in FOSC isolates because no suitable primers were available. Gene expression levels were normalised against the housekeeping gene *GPD* amplified with primers NkGPD1‐829‐F (5′‐CCTTCTGGGAGCAGCTTAAA‐3′) and NkGPD1‐1050‐R (5′‐TGAAGAGAGCAACCGAAATACA‐3′) [17, 34].

Quantitative PCR assays were performed using SYBR Green technology on a Step One Plus Real‐Time PCR System (Applied Biosystems, USA) with the qPCRBIO SyGreen Mix Separate‐ROX kit (PCR Biosystems), according to the manufacturer's instructions. Primer efficiency was determined using a genomic DNA dilution series, and only primer sets with amplification efficiencies between 95% and 105% were considered acceptable. Primer performance data are described in Table S2. Relative expression levels were calculated using the 2^−ΔΔCq^ method [35]. Expression of *CYP51A* and *ABC1* was evaluated by comparing VOR‐exposed and non‐exposed cultures. All experiments were performed with three biological replicates and technical triplicates. Results are presented as mean ± SD calculated from technical triplicates. One representative experiment was selected for graphical presentation. Mean ± SD from all biological replicates were compiled for comparative assessment.

## 3 Results

### 3.1 Molecular Identification and Phylogenetic Analyses

**FIGURE 1:**
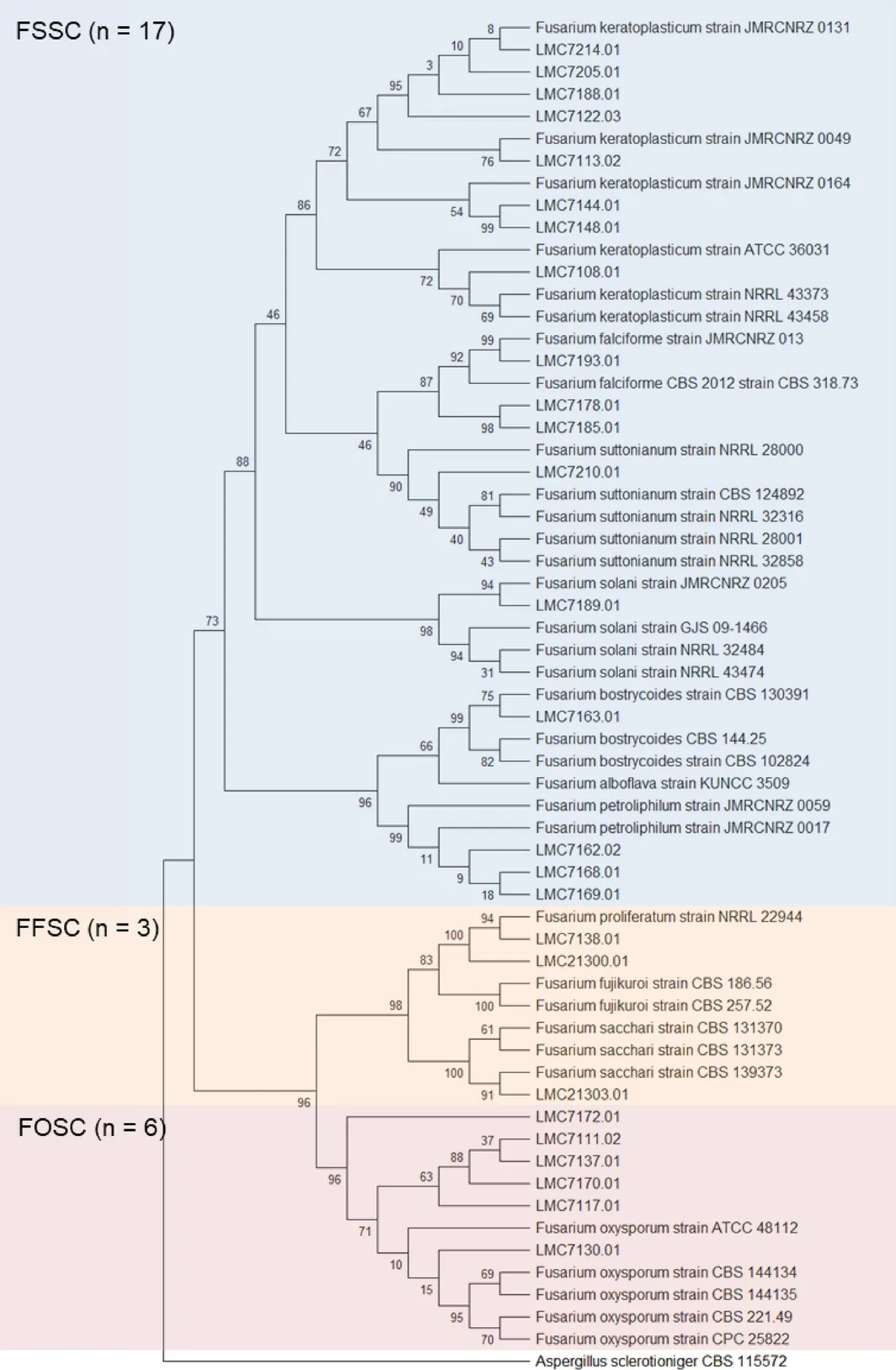
Species‐complex level phylogenetic placement of *Fusarium* clinical isolates inferred from concatenated *TEF1* and *RPB2* sequences. Maximum‐likelihood phylogenetic tree showing species‐complex level clustering of *Fusarium* clinical isolates based on concatenated *TEF1* and *RPB2* sequences. The analysis included 26 clinical isolates, two reference strains and additional reference sequences representing clinically relevant *Fusarium* taxa. The tree resolved three major groups corresponding to the *Fusarium solani* species complex (FSSC), *Fusarium fujikuroi* species complex (FFSC) and *Fusarium oxysporum* species complex (FOSC). Clinical isolates assigned to the FOSC grouped with *F. oxysporum* reference sequences, whereas FFSC isolates clustered with *F. proliferatum* and *F. sacchari*. Within the FSSC, the topology supported the placement of isolates in a broader FSSC cluster, with finer species‐level resolution addressed in Figure 2. Bootstrap values based on 1000 replicates are shown at the nodes. *Aspergillus sclerotiorum* CBS 115572 was used as the outgroup. The final dataset comprised 61 nucleotide sequences and 1621 aligned positions.

A total of 26 *Fusarium* clinical isolates and two reference strains were analysed by sequencing of the ITS region, *TEF1* and *RPB2* loci. Initial ITS‐based analysis assigned the clinical isolates to three major species complexes: 17 clinical isolates belonged to the FSSC, 6 to the FOSC and 3 to the FFSC (Table S3). Phylogenetic analysis based on concatenated *TEF1* and *RPB2* sequences confirmed the distribution and supported the identification of all FOSC isolates as *F. oxysporum* and FFSC isolates as *F. proliferatum* (*n* = 2) and *F. sacchari* (*n* = 1) (Figure 1).

**FIGURE 2:**
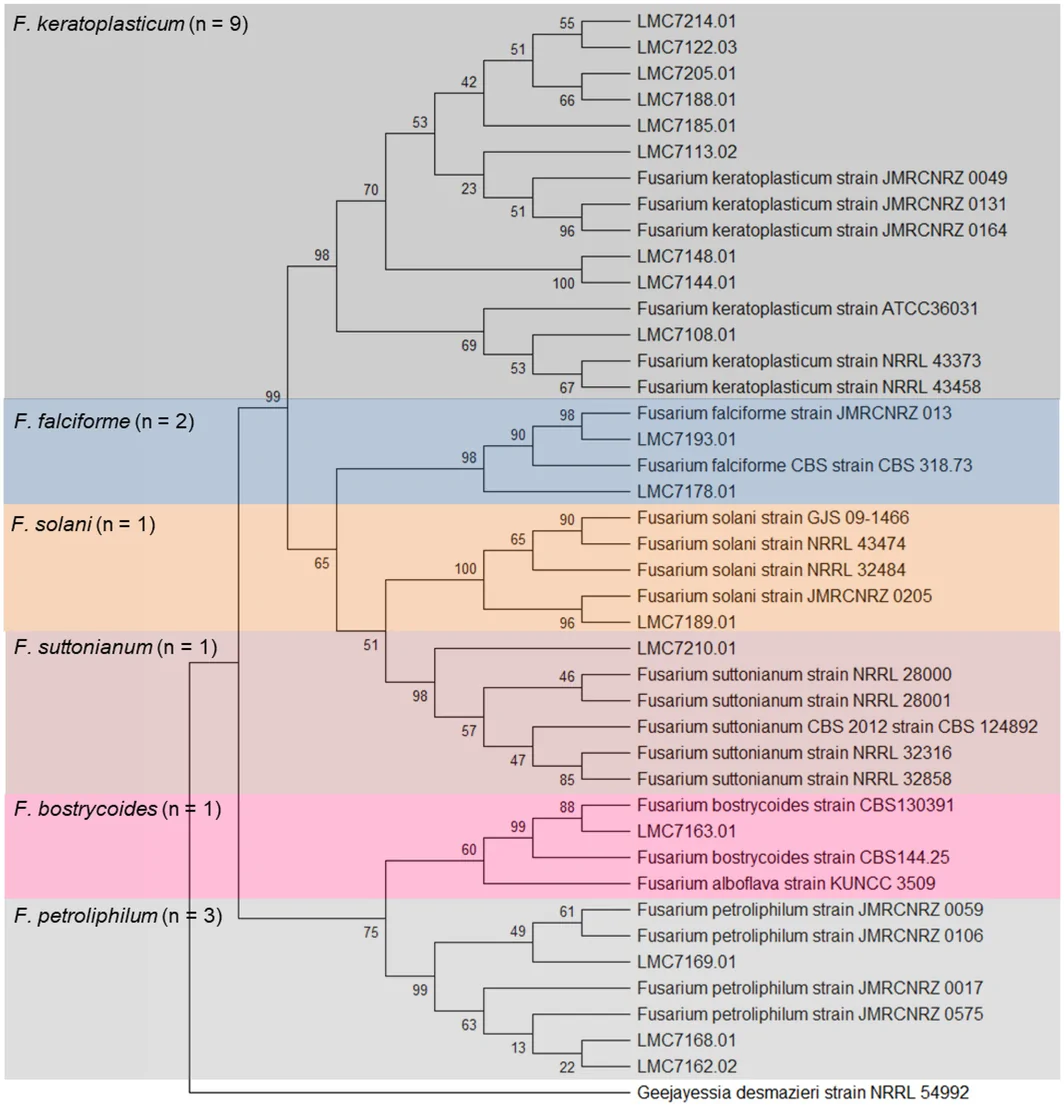
Maximum‐likelihood phylogenetic tree showing species‐level resolution within the *Fusarium solani* species complex (FSSC) based on concatenated ITS, *TEF1* and *RPB2* sequences. Maximum‐likelihood phylogenetic tree showing species‐level resolution within the *Fusarium solani* species complex based on concatenated ITS, *TEF1* and *RPB2* sequences. The analysis included 17 clinical isolates assigned to the FSSC and additional reference sequences representing *F. keratoplasticum*, *F. falciforme, F. solani, F. suttonianum, F. bostrycoides* and *F. petroliphilum*. The tree supported the identification of the clinical isolates as *F. keratoplasticum* (*n* = 9), *F. petroliphilum* (*n* = 3), *F. falciforme* (*n* = 2), *F. solani* (*n* = 1), *F. suttonianum* (*n* = 1) and *F*. *bostrycoides* (*n* = 1). Bootstrap values based on 1000 replicates are shown at the nodes. *Geejayessia desmazieri* strain NRRL 54992 was used as the outgroup. The final dataset comprised 42 nucleotide sequences and 2027 aligned positions.

Because species‐level delimitation within the FSSC remained limited using the concatenated *TEF1* and *RPB2* dataset, a second analysis including the ITS was performed, supporting the identification of the 17 FSSC clinical isolates as *F. keratoplasticum* (*n* = 9), *F. petroliphilum* (*n* = 3), *F. falciforme* (*n* = 2), *F. solani* (*n* = 1), *F. suttonianum* (*n* = 1) and *F. bostrycoides* (*n* = 1) (Figure 2).

### 3.2 Antifungal Susceptibility Profiles

The antifungal susceptibility of 26 *Fusarium* spp. clinical isolates was evaluated against amphotericin B (AMB), voriconazole (VOR), itraconazole (ITR) and posaconazole (POS). Overall, the isolates showed elevated MIC values for most antifungal agents tested, consistent with the intrinsically low susceptibility commonly observed in clinically relevant *Fusarium* species complexes. Among the four drugs evaluated, ITR exhibited the poorest in vitro activity, with all isolates showing MIC values above the tested range limit (> 32 μg/mL). By contrast, AMB showed the lowest MIC values overall, although one FSSC isolate displayed a markedly elevated MIC (> 32 μg/mL). Voriconazole and POS showed variable activity across species complexes, with broader MIC distributions observed mainly among FSSC isolates (Table 1, Table S4).

**TABLE 1:** In vitro antifungal susceptibility of clinical and reference *Fusarium* isolates.

Species complex	Isolate/strain	Species	AMB	VOR	ITR	POS
FSSC	LMC7163.01	*F. bostrycoides*	1	32	> 32^a^	> 32^a^
	LMC7178.01	*F. falciforme*	2	16	> 32^a^	> 32^a^
	LMC7193.01	*F. falciforme*	2	32	> 32^a^	> 32^a^
	LMC7108.01	*F. keratoplasticum*	> 32^a^	8	> 32^a^	> 32^a^
	LMC7113.02	*F. keratoplasticum*	2	> 32^a^	> 32^a^	> 32^a^
	LMC7122.03	*F. keratoplasticum*	2	> 32^a^	> 32^a^	> 32^a^
	LMC7144.01	*F. keratoplasticum*	2	2	> 32^a^	> 32^a^
	LMC7148.01	*F. keratoplasticum*	2	2	> 32^a^	> 32^a^
	LMC7185.01	*F. keratoplasticum*	2	8	> 32^a^	> 32^a^
	LMC7188.01	*F. keratoplasticum*	2	8	> 32^a^	> 32^a^
	LMC7205.01	*F. keratoplasticum*	4	> 32^a^	> 32^a^	> 32^a^
	LMC7214.01	*F. keratoplasticum*	2	> 32^a^	> 32^a^	> 32^a^
	LMC7162.02	*F. petroliphilum*	2	32	> 32^a^	> 32^a^
	LMC7168.01	*F. petroliphilum*	1	4	> 32^a^	> 32^a^
	LMC7169.01	*F. petroliphilum*	2	> 32^a^	> 32^a^	> 32^a^
	LMC7189.01	*F. solani*	1	2	> 32^a^	> 32^a^
	LMC7210.01	*F. suttonianum*	2	> 32^a^	> 32^a^	> 32^a^
	ATCC 36031	*F. keratoplasticum*	2	16	> 32^a^	> 32^a^
FFSC	LMC21300.01	*F. proliferatum*	2	8	> 32	> 32
	LMC7138.01	*F. proliferatum*	2	4	> 32	2
	LMC21303.01	*F. sacchari*	2	4	> 32	> 32
FOSC	LMC7111.02	*F. oxysporum*	2	2	> 32^a^	> 32^a^
	LMC7117.01	*F. oxysporum*	2	4	> 32^a^	> 32^a^
	LMC7130.01	*F. oxysporum*	2	8	> 32^a^	> 32^a^
	LMC7137.01	*F. oxysporum*	2	4	> 32^a^	2
	LMC7170.01	*F. oxysporum*	2	2	> 32^a^	4
	LMC7172.01	*F. oxysporum*	2	4	> 32^a^	> 32^a^
	ATCC 48112	*F. oxysporum*	2	16	> 32^a^	> 32^a^

*Note:* Antifungal concentration range tested: 0.06–32 μg/mL.

Abbreviations: AMB, amphotericin B; FFSC, *Fusarium fujikuroi* species complex; FOSC, *Fusarium oxysporum* species complex; FSSC, *Fusarium solani* species complex; ITR, itraconazole; MIC, minimum inhibitory concentration; POS, posaconazole; VOR, voriconazole.

^a^ MIC value above the epidemiological cutoff value (ECV) proposed for the corresponding species complex, and therefore classified as non‐wild‐type (NWT).

Because no clinical breakpoints have been established for *Fusarium* spp., susceptibility profiles were interpreted using epidemiological cutoff values (ECV), allowing classification as wild‐type (WT) or non‐wild‐type (NWT) [11]. Based on these criteria, one FSSC isolate was classified as NWT for AMB, 23 isolates were classified as NWT for POS (17 FSSC and 6 FOSC) and 6 FSSC isolates were classified as NWT for VOR. For ITR, all isolates showed MIC values above the tested range, indicating a uniformly non‐wild‐type profile (Table 1 and Table S4).

**FIGURE 3:**
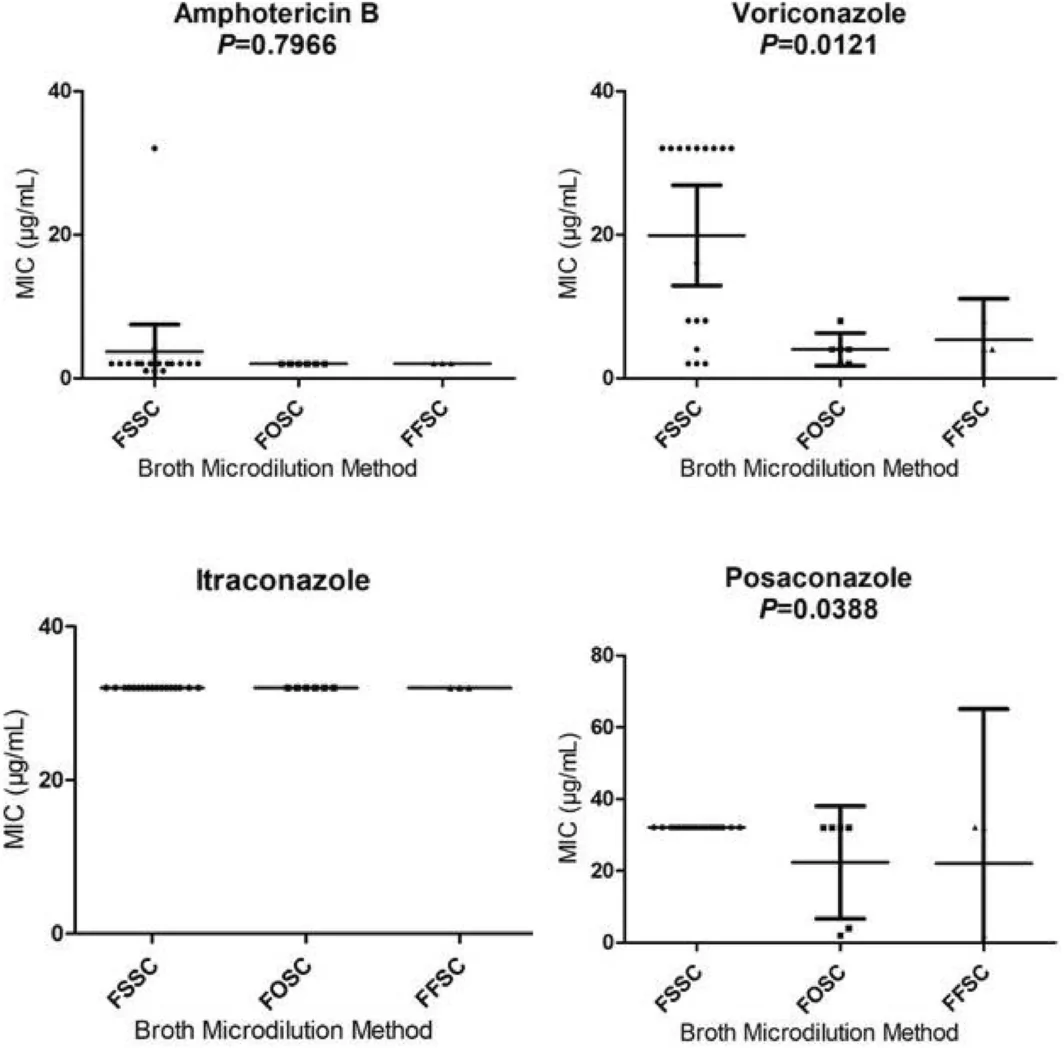
Comparison of antifungal MIC distributions among clinical isolates of the FSSC, FOSC and FFSC. Distribution of MIC values for amphotericin B (AMB), voriconazole (VOR), itraconazole (ITR) and posaconazole (POS) among clinical isolates belonging to the FSSC (*n* = 17), FOSC (*n* = 6) and FFSC (*n* = 3). Bars represent mean ± standard deviation. Statistical comparisons among species complexes were performed using a one‐way test, and *p* values are shown in the corresponding panels. Significant differences were observed for VOR and POS, whereas no significant difference was detected for AMB. Interpretation of ITR comparisons was limited because MIC values were uniformly high (> 32 μg/mL) across all groups.

Analysis by species complex showed that FSSC isolates had the highest MIC values overall, particularly for VOR and POS. In contrast, FOSC and FFSC isolates tended to show lower VOR MICs than FSSC isolates. Statistical analysis revealed differences among species complexes for VOR (*p* = 0.0121) and POS (*p* = 0.0388), whereas no significant differences were observed for AMB (*p* = 0.7966). Because ITR MICs were uniformly high across all groups, comparative statistical interpretation for this drug was limited (Figure 3). Together, these findings indicate that *Fusarium* clinical isolates display high MIC values to azoles overall, with the FSSC showing the least favourable susceptibility profile among the complexes analysed.

### 3.3 In Silico Identification of *CYP51A* Reference Sequences and Primer Design

To extend *CYP51A* amplification across the three species complexes included in this study, representative reference sequences from the FSSC, FOSC and FFSC were analysed in silico to identify conserved regions suitable for primer annealing. Phylogenetic analysis of *CYP51A* sequences by the UPGMA method supported clustering at the species‐complex level, and subsequent alignments performed separately for the FSSC, FOSC and FFSC allowed the identification of conserved regions for primer design (Table S5, Figure S1). Based on the analyses, primer sets were established for *CYP51A* amplification and sequencing across the three complexes (Table 2), enabling the subsequent comparative sequence analysis of the clinical isolate panel.

**TABLE 2:** Primers designed in this study for *CYP51A* amplification and sequencing across FSSC, FOSC and FFSC.

Species complex	Position relative to the *CYP51A* start codon (bp)	Direction	Primer name	Sequence 5′ → 3′
FSSC	−426	Forward	FSSC1	AAAATGCTAAGCGAACCCTGC
	−159	Forward	FSSC2	CAGAGTGTTGGAGGATGAAG
	+116	Forward	FSSC3	CACTGGCTTCCCTTCATCG
	+313	Reverse	FSSC4	CTCACAACCGAAAACGAGAC
	+736	Forward	FSSC5	AAAGTCGTTGCCTTCGACTC
	+842	Reverse	FSSC6	CCATCAGGTTGGCAATCATG
	+1593	Reverse	FSSC7	TAACAGCTTCATGCCTTGCG
FOSC	−399	Forward	FOSC1	TGATTCAGGCTTACGATCGG
	+2	Forward	FOSC2	TGTTCTCACTCCTATACTACC
	+162	Reverse	FOSC3	GCTACCTTTGCCTCATAACC
	+716	Forward	FOSC4	GTCGAGTCCATAAGCAACAG
	+844	Reverse	FOSC5	TTGGCGATCATGTCAGTTCC
	+1527	Reverse	FOSC6	TATGTATGCAGGTTGGGCTG
FFSC	+1	Forward	FFSC1	ATGTTTCCACTCCTCTACTAC
	+723	Forward	FFSC2	CATGGACATTATCAACGAACG
	+901	Reverse	FFSC3	TCGCAATTCATCAGGTTGGC
	+1520	Reverse	FFSC4	TCAAGCCTTCCTGCGTTGC

*Note:* Primer positions are shown relative to the *CYP51A* start codon. Negative values indicate upstream positions and positive values indicate positions within the coding sequence.

Abbreviations: FFSC, *Fusarium fujikuroi* species complex; FOSC, *Fusarium oxysporum* species complex; FSSC, *Fusarium solani* species complex.

**FIGURE 4:**
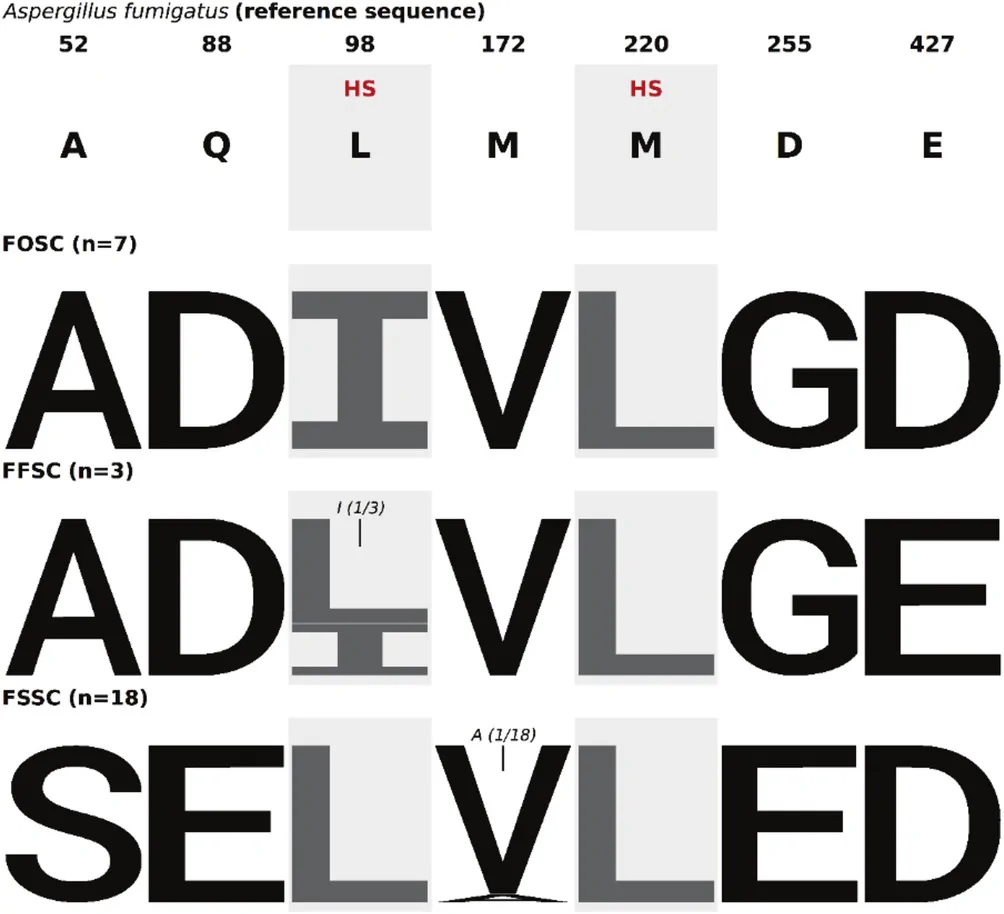
CYP51A amino acid signatures at positions equivalent to *Aspergillus fumigatus* in clinical isolates and reference strains of the FSSC, FOSC and FFSC. Amino acid profiles are shown for residues equivalent to positions 52, 88, 98, 172, 220, 255 and 427 of *Aspergillus fumigatus* Cyp51A among clinical isolates of the FOSC (*n* = 7), FFSC (*n* = 3) and FSSC (*n* = 18). All amino acid positions were examined in the sequence comparison. The residues shown here represent polymorphic sites that distinguish the *Fusarium* species complexes, including positions equivalent to 98 and 220 in *A. fumigatus* , indicated as HS (canonical azole‐resistance hotspot‐equivalent sites), because they correspond to well‐characterised azole‐resistance‐associated positions in that species. The top row shows the reference sequence of *A. fumigatus* . Residue numbering is based on positions equivalent to *A. fumigatus* Cyp51A. Annotations such as I (1/3) and A (1/18) indicate minor variants within a species complex, where the amino acid shown was detected in 1 isolate out of the total number of sequences analysed for that group.

The *CYP51A* gene was successfully amplified and sequenced in the 26 *Fusarium* spp. clinical isolates using the primer sets designed for each species complex. The resulting coding sequences were analysed at both nucleotide and amino acid levels, and the amino acid states identified in each isolate are summarised in Table S6. For amino acid comparison, each *Fusarium* CYP51A sequence was aligned using the well‐characterised *Aspergillus fumigatus* Cyp51A as the positional reference framework for examination of all amino acid positions. Among the polymorphic sites identified, residues equivalent to positions 98 and 220 were of particular interest because alterations at these positions in *A. fumigatus* have been associated with structural effects on the ligand access channel, thereby affecting azole access to the catalytic region of the enzyme [36]. Distinct amino acid profiles were observed among the three *Fusarium* species complexes. FSSC isolates showed the pattern S52/E88/L98/V172/L220/E255/D427, with minor variation at position 172 in *F. falciforme*. FFSC isolates showed A52/D88/L98/V172/L220/G255/E427, whereas FOSC isolates showed A52/D88/I98/V172/L220/G255/D427 (Figure 4, Table S6).

Despite these sequence differences, no clear association was observed between CYP51A amino acid profiles and azole MIC values in the present isolate collection. In particular, isolates sharing the same amino acid pattern frequently displayed distinct voriconazole and posaconazole MICs, indicating that variation in CYP51A sequence alone was insufficient to explain the phenotypic diversity in azole susceptibility observed here. Thus, although residues equivalent to positions 98 and 220 in *A. fumigatus* were noteworthy because of their established relevance in azole resistance studies, the additional polymorphic residues shown also contributed to the species‐complex‐associated amino acid signatures identified in this study (Figure 4, Table S6).

**FIGURE 5:**
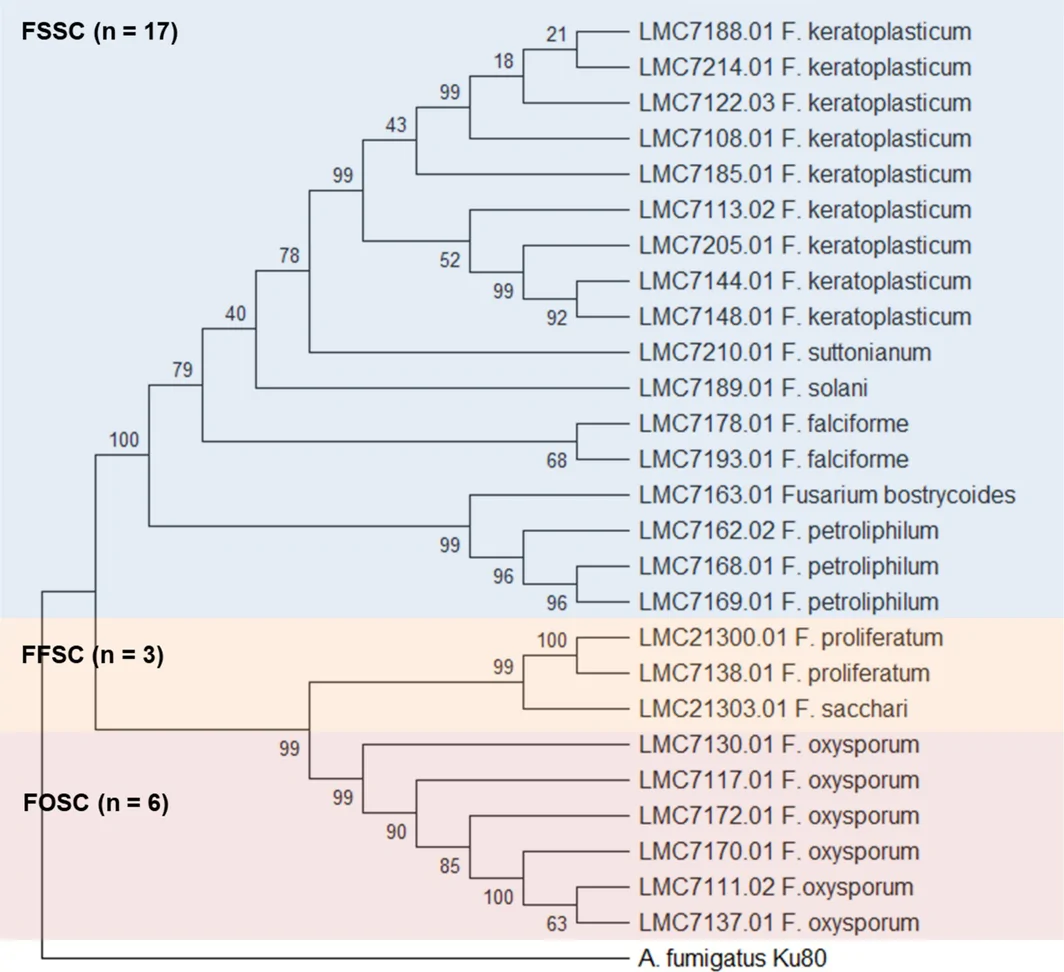
Maximum‐likelihood phylogenetic clustering of *Fusarium* spp. clinical isolates based on CYP51A coding sequences. Maximum‐likelihood phylogenetic tree inferred from CYP51A coding sequences of 26 *Fusarium* clinical isolates, showing clustering into the *Fusarium solani* species complex (FSSC; *n* = 17), *Fusarium fujikuroi* species complex (FFSC; *n* = 3) and *Fusarium oxysporum* species complex (FOSC; *n* = 6). Bootstrap values from 1000 replicates are shown at the nodes. The final alignment comprised 27 nucleotide sequences and 1548 aligned positions. *Aspergillus fumigatus* (ΔakuB^KU80^ strain) was used as the outgroup.

Phylogenetic analysis based on CYP51A coding sequences resolved three major clades corresponding to the *Fusarium solani* species complex (FSSC), *Fusarium fujikuroi* species complex (FFSC) and *Fusarium oxysporum* species complex (FOSC) (Figure 5). Within the FSSC, the topology was consistent with the species assignments previously obtained by multilocus identification, including the separation of *F. keratoplasticum, F. petroliphilum, F. falciforme, F. solani, F. suttonianum* and *F. bostrycoides*. The FFSC clade included the isolates identified as *F. proliferatum* and *F. sacchari*, whereas the FOSC clade grouped all *F. oxysporum* isolates. Thus, in addition to its value for comparative sequence analysis, CYP51A retained sufficient phylogenetic signal to discriminate the major *Fusarium* species complexes represented in this study and to reflect species‐level relationships within some of these groups.

### 3.4 *CYP51A* and *ABC1* Expression in Response to Voriconazole Exposure

Selected isolates represented distinct voriconazole susceptibility profiles within the FSSC and FOSC. In the FSSC, *F. keratoplasticum* LMC7108.01 (VOR MIC = 8 μg/mL) and LMC7113.02 (VOR MIC > 32 μg/mL) were selected to represent lower and higher voriconazole MIC values, respectively, together with the reference strain ATCC 36031 (VOR MIC = 16 μg/mL). In the FOSC, *F. oxysporum* LMC7137.01 (VOR MIC = 4 μg/mL) and the reference strain ATCC 48112 (VOR MIC = 16 μg/mL) were selected to represent lower and higher voriconazole MIC values, respectively. Expression of *CYP51A* and *ABC1* was evaluated after exposure to voriconazole at 8 μg/mL for 6 h. All primer sets used for RT‐qPCR showed amplification efficiencies within the acceptable range (95%–105%) (Table S2), thereby supporting the reliability of the RT‐qPCR results. The same overall expression trend was observed across the biological replicates, and the fold‐change values obtained in the three biological replicates, expressed as mean ± SD of technical triplicates, are summarised in Table S6.

**FIGURE 6:**
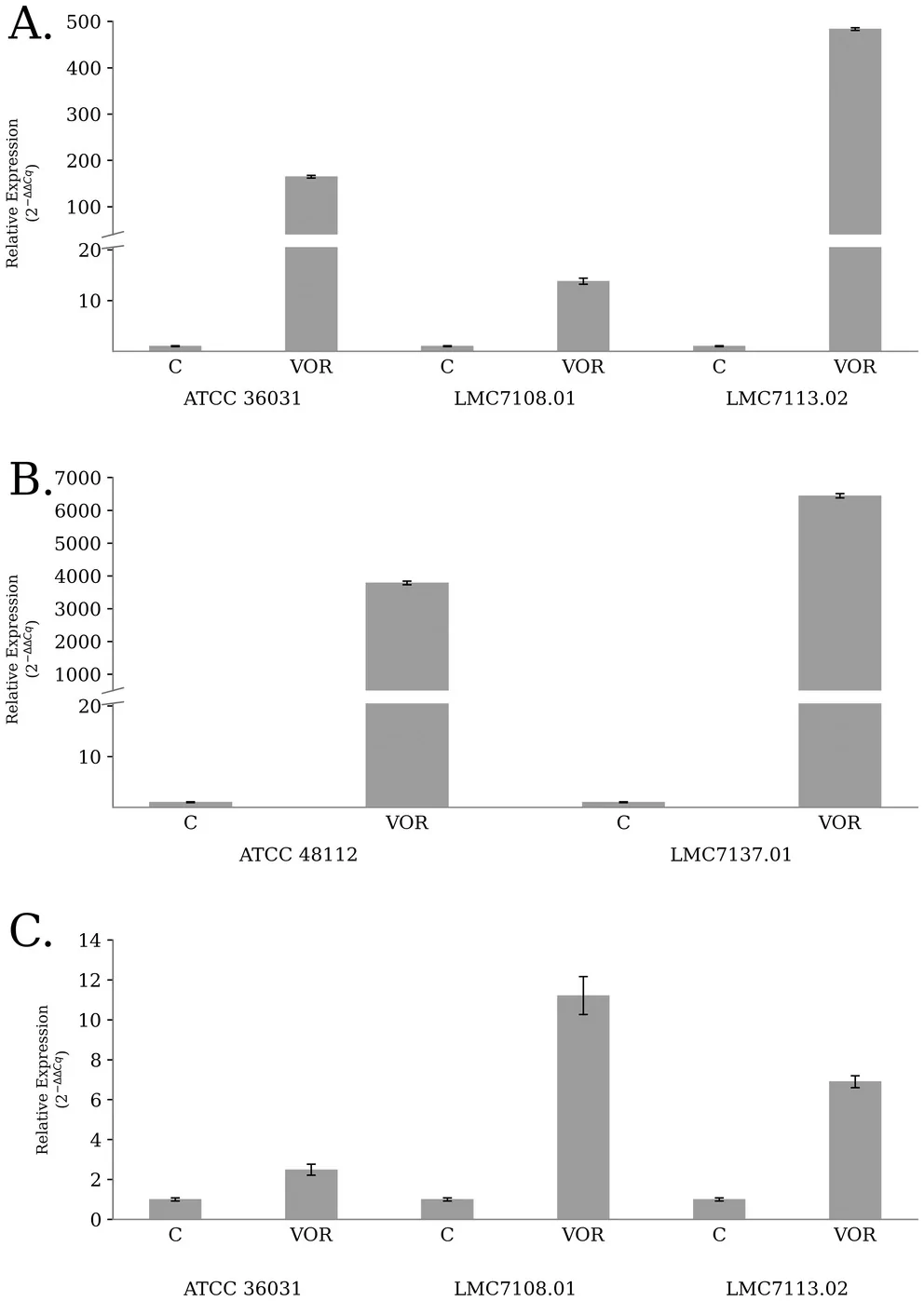
Transcriptional response of *CYP51A* and *ABC1* to voriconazole exposure in selected *Fusarium* isolates. (A) Relative *CYP51A* expression in FSSC isolates (*F. keratoplasticum* ATCC 36031, LMC7108.01 and LMC7113.02). (B) Relative *CYP51A* expression in FOSC isolates (*F. oxysporum* ATCC 48112 and LMC7137.01). (C) Relative *ABC1* expression in FSSC isolates (*F. keratoplasticum* ATCC 36031, LMC7108.01 and LMC7113.02). Cultures were exposed to voriconazole (8 μg/mL) at 30°C and 150 rpm for 6 h, and expression levels were normalised to the corresponding untreated controls. Relative expression was calculated by the 2^−ΔΔCq^ method. The panels shown correspond to one representative experiment. Fold‐change values for all biological replicates, expressed as mean ± SD of technical triplicates, are summarised in Table S7.

Exposure to voriconazole induced marked upregulation of *CYP51A* in all isolates analysed. Among FSSC isolates, relative expression ranged from 13.8‐fold to 483.5‐fold in the representative experiment shown in Figure 6A, with the highest induction observed in LMC7113.02, followed by ATCC 36031 and LMC7108.01 (Figure 6A). Notably, a marked difference in *CYP51A* induction was observed between the two clinical FSSC isolates, with the isolate showing the higher voriconazole MIC (LMC7113.02; VOR MIC > 32 μg/mL) also displaying substantially higher expression than LMC7108.01 (VOR MIC = 8 μg/mL). In FOSC, *CYP51A* expression was also strongly induced, with relative expression values of 3785‐fold in ATCC 48112 and 6443‐fold in LMC7137.01 in the representative experiment shown in Figure 6B. Overall, the magnitude of *CYP51A* induction was observed in both species complexes, with higher expression levels in FOSC and marked variation among isolates within the FSSC under the conditions tested.

Expression of the efflux pump gene *ABC1* was evaluated in the FSSC isolates and reference strain. In this group, *ABC1* was also upregulated following voriconazole exposure, with relative expression values ranging from 2.49‐fold to 11.21‐fold in the representative experiment shown in Figure 6C. However, the magnitude of *ABC1* induction was lower than that observed for CYP51A in the same isolates, indicating a stronger transcriptional response of the target‐related gene than of the efflux‐associated gene under voriconazole exposure.

## 4 Discussion

In this study, 26 clinical isolates of *Fusarium* spp. from patients in the State of São Paulo, Brazil, were characterised by multilocus sequencing and grouped into the FSSC, FOSC and FFSC. As observed in previous clinical studies, the FSSC was the most frequent complex in the present collection, reinforcing its recognised predominance among clinically relevant *Fusarium* isolates and its central role in human fusariosis [3, 37]. This taxonomic distribution is clinically relevant since susceptibility profiles and resistance‐associated mechanisms may vary among species complexes and even among isolates within the same complex.

Antifungal susceptibility testing confirmed the overall reduced in vitro activity of azoles against the analysed isolates, particularly within the FSSC, which showed the least favourable susceptibility profile among the three complexes. Itraconazole exhibited uniformly high MIC values, whereas voriconazole and posaconazole showed broader variability, especially among FSSC isolates. Amphotericin B showed the lowest MICs overall, although one FSSC isolate displayed an elevated MIC. These findings are consistent with previous reports describing intrinsically low susceptibility of *Fusarium* spp. to multiple antifungal classes, including azoles, polyenes and echinocandins, and reinforce the therapeutic difficulty posed by these infections [3, 6, 7, 38]. Because clinical breakpoints are not available for *Fusarium* spp., the use of epidemiological cutoff values (ECVs) provides a useful framework for surveillance and for identifying isolates with non‐wild‐type profiles, although these values should not be interpreted as direct predictors of clinical outcome [11]. In this context, the high proportion of isolates classified as non‐wild‐type for posaconazole and the uniformly elevated MICs observed for itraconazole further emphasise the limited activity of triazoles against these organisms.

The reduced susceptibility of *Fusarium* spp. to azoles is unlikely to depend on a single mechanism. Current evidence in *Fusarium* supports a multifactorial framework involving target alterations, overexpression of *CYP51* genes, drug efflux, and, in some settings, biofilm‐associated tolerance mechanisms [15]. This broader view is also consistent with recent discussions emphasising the complexity of azole resistance in *Fusarium* and the limited molecular evidence currently available for clearly defining resistance markers in clinical isolates [39, 40]. In this context, the present study explored this multifactorial scenario through a combination of *CYP51A* sequence analysis and transcriptional profiling of *CYP51A* and *ABC1* under voriconazole stress.

Comparative analysis of CYP51A amino acid signatures revealed distinct profiles among the FSSC, FOSC and FFSC, confirming that this gene carries informative variation at the species‐complex level. At the same time, the phylogenetic analysis based on CYP51A coding sequences resolved the three major complexes and was broadly consistent with the species assignments obtained by multilocus identification, indicating that CYP51A retains sufficient phylogenetic signal to discriminate clinically relevant *Fusarium* groups. Nevertheless, although CYP51A sequence variation clearly reflected taxonomic structure, no straightforward association was observed between amino acid profiles and azole MIC values in the isolate collection analysed here. Isolates sharing the same amino acid pattern frequently displayed different voriconazole and posaconazole MICs, indicating that CYP51A sequence alone was insufficient to explain the phenotypic diversity in azole susceptibility observed in this study.

To interpret these findings in a broader mechanistic context, *Fusarium* CYP51A sequences were compared with the well‐characterised *Aspergillus fumigatus* Cyp51A reference sequence from a wild‐type (azole‐susceptible strain), used here as the baseline for positional comparison [12, 14, 36, 41]. This comparison showed that residues corresponding to positions 98 and 220 in *A. fumigatus* , both recognised as major hotspot positions in azole resistance studies, were also represented among the *Fusarium* sequences analysed here. In particular, FOSC isolates exhibited isoleucine at the position equivalent to L98, while a conserved leucine was observed at the position corresponding to M220 across multiple *Fusarium* species complexes, including FSSC, FOSC and FFSC. These observations are noteworthy because substitutions affecting L98 and M220 in *A. fumigatus* have been repeatedly associated with reduced azole susceptibility and resistant phenotypes, with structural effects involving the ligand access channel and the azole‐binding environment of the enzyme [14, 36, 41, 42, 43]. In addition, Vermeulen et al. [3] proposed that equivalent residues in FSSC CYP51 proteins may contribute to the intrinsically low azole susceptibility of *Fusarium*, particularly because these sequence features are conserved across strains with generally reduced susceptibility profiles.

However, the present data do not support a simple relationship between CYP51A amino acid variation and azole MIC values in *Fusarium*. Although residues equivalent to canonical *A. fumigatus* hotspot positions were identified, their presence alone was not sufficient to explain the variation in azole MIC values observed among isolates. These residues should therefore be interpreted as candidate structural features that may contribute to the intrinsically reduced azole susceptibility of *Fusarium*, rather than as independent determinants of resistance. In this regard, our findings are broadly consistent with previous observations that *Fusarium* CYP51A may harbour conserved substitutions relative to *A. fumigatus* , while also indicating that their contribution is better understood within a broader resistance framework than as a single explanatory mechanism [3]. Likewise, substitutions such as S52A, Q88D/E, M172V, D255E/G and E427D appeared to define lineage‐associated amino acid signatures rather than clear markers of high‐MIC phenotypes, consistent with the view that some peripheral substitutions may have limited or context‐dependent functional impact [3, 14, 41].

A more complete explanation of azole response emerges when the expression data are considered. One of the most relevant findings of this study was the marked induction of *CYP51A* under voriconazole exposure. Upregulation of *CYP51A* was observed in all tested isolates, including representatives of both the FSSC and FOSC, indicating that transcriptional activation of the target gene is a robust response to azole stress in these complexes. This observation is especially relevant because the magnitude of induction varied markedly among isolates. Within the FSSC, LMC7113.02, which showed the highest voriconazole MIC among the tested isolates, also displayed greater *CYP51A* expression than LMC7108.01, which had a lower voriconazole MIC. This observation suggests that target‐gene induction may contribute to differences in azole response in selected isolates, although this interpretation should be made cautiously because only a limited number of strains were included in the induction experiments. Even so, the data support the view that transcriptional regulation of *CYP51A* may represent a biologically meaningful component of azole adaptation in *Fusarium*. This interpretation is consistent with previous work showing that azole exposure can induce *CYP51A* overexpression in *Fusarium* isolates. D'Agostino et al. [16], for example, reported marked induction of *CYP51A* in FSSC isolates under azole exposure, suggesting that target‐gene overexpression may contribute to reduced susceptibility. Similar observations have also been reported in agricultural *Fusarium* species exposed to demethylase inhibitors, supporting the idea that inducible *CYP51A* transcription is not restricted to clinical settings and may reflect a broader adaptive strategy of the genus [44]. In this context, the strong induction observed here under voriconazole exposure reinforces the importance of considering not only constitutive sequence variation but also the dynamic transcriptional behaviour of the target gene.

In contrast to the pronounced induction of *CYP51A*, the efflux‐associated gene *ABC1* showed only modest upregulation in the FSSC isolates analysed. This result does not exclude a role for efflux‐mediated mechanisms, but suggests that, under the experimental conditions used here, the transcriptional response of the target‐related gene was more prominent than that of the tested efflux transporter. This interpretation is compatible with previous studies showing that ABC transporters can contribute to azole response and virulence in different *Fusarium* species, while also indicating that their relative contribution may vary according to species, isolate and experimental context [17, 45, 46]. In *F. keratoplasticum*, James et al. [17] demonstrated that the ATP‐binding cassette transporter *ABC1* contributes to innate azole resistance, supporting the idea that efflux should remain part of the mechanistic framework. However, the relatively lower induction of *ABC1* observed in the present study suggests that, at least in the tested FSSC isolates and under the applied voriconazole conditions, *CYP51A* overexpression was the dominant detectable transcriptional response.

Taken together, our data support a multifactorial model of azole response in *Fusarium* spp. Reduced susceptibility appears to involve conserved CYP51A sequence features, marked induction of the target gene under voriconazole exposure, and, to a lesser extent, efflux‐associated mechanisms. This framework also explains why amino acid signatures alone did not segregate neatly with MIC phenotypes in the present isolate collection.

These findings should also be considered in a broader environmental context. The extensive use of azole fungicides in agriculture has been discussed as a source of selection pressure capable of shaping fungal populations with reduced susceptibility to medical triazoles, particularly in trans‐kingdom pathogens such as *Fusarium* [39]. Thus, the mechanisms identified here may be relevant not only to clinical isolates but also within a wider One Health framework linking environmental exposure, agricultural fungicide use and human infection.

Overall, this study expands current knowledge of azole resistance‐related mechanisms in clinically relevant *Fusarium* species complexes by integrating multilocus identification, susceptibility testing, *CYP51A* sequence analysis and gene expression profiling. The results support the interpretation of azole resistance in *Fusarium* as a multifactorial phenotype and reinforce the need for continued molecular surveillance, improved susceptibility monitoring and mechanistically informed therapeutic strategies.

## 5 Conclusion

In conclusion, this study supports a multifactorial model of azole response in clinically relevant *Fusarium* species complexes. Conserved CYP51A amino acid features, strong induction of *CYP51A* under voriconazole exposure and lower but detectable *ABC1* upregulation support the view that reduced azole susceptibility in *Fusarium* is unlikely to depend on a single mechanism. Rather, it appears to result from the interplay between constitutive sequence background and inducible adaptive responses. These findings expand current knowledge of azole resistance‐related mechanisms in *Fusarium* and highlight the need for continued molecular surveillance and improved antifungal susceptibility monitoring.

## Author Contributions

**Patrícia Helena Grizante Barião:** methodology, data curation, investigation, formal analysis, writing – review and editing, writing – original draft, validation, visualization. **Adriana Rodrigues Alves:** methodology. **Otávio Guilherme Gonçalves de Almeida:** methodology. **Cledir Santos:** conceptualization, funding acquisition, writing – review and editing. **Marcia Regina von Zeska Kress:** conceptualization, funding acquisition, writing – original draft, writing – review and editing, supervision, project administration, formal analysis, resources. **Ludmilla Tonani:** methodology, formal analysis, validation, writing – review and editing.

## Funding

The Article Process Charge for the publication of this research was funded by the Coordination for the Improvement of Higher Education Personnel—CAPES (ROR identifier: 00x0ma614). This study was financed in part by the ‘Coordenação de Aperfeiçoamento de Pessoal de Nível Superior’—Brazil (CAPES)—Finance Code 001. This research was partially funded by the ‘Fundação de Amparo à Pesquisa do Estado de São Paulo’ (FAPESP, Brazil) and the Universidad de La Frontera (UFRO, Chile) through the project FAPESP‐UFRO 2020/07546‐2, and by FAPESP through the project 2023/12463‐7. The authors also acknowledge funding from the Conselho Nacional de Desenvolvimento Científico e Tecnológico (CNPq, Brazil) (310425/2021‐2 and 304351/2025‐3).

## Conflicts of Interest

The authors declare no conflicts of interest.

## Supporting information


**Figure S1:** UPGMA tree of *CYP51A* reference sequences used for primer design across the FSSC, FOSC and FFSC. UPGMA tree inferred from *CYP51A* reference sequences used for primer design across the *Fusarium solani* species complex (FSSC), *Fusarium oxysporum* species complex (FOSC) and *Fusarium fujikuroi* species complex (FFSC). The tree shows clustering of the selected sequences according to species‐complex level similarity, supporting the identification of conserved regions for subsequent alignment and primer design. Bootstrap values are shown at the nodes, and the scale bar represents genetic distance. Ffal, *F. falciforme*; Fke, *F. keratoplasticum*; Fpro, *F. proliferatum*; Ffu, *F. fujikuroi*; Fox, *F. oxysporum*.
**Table S1:** Clinical isolates of *Fusarium* spp. and their biological sites of isolation. FSSC, *F. solani* species complex; FFSC, *F. fujikuroi* species complex; FOSC, *F. oxysporum* species complex.
**Table S2:** Primer sets used for RT‐qPCR and amplification efficiencies. FSSC, *F. solani* species complex; FOSC, *F. oxysporum* species complex.
**Table S3:** BLAST‐based molecular identification of *Fusarium* clinical isolates using ITS, *TEF1* and *RPB2* sequences, including GenBank reference accessions and GenBank accession numbers of sequences generated in this study. FSSC, *Fusarium solani* species complex; FFSC, *Fusarium fujikuroi* Species complex; FOSC, *Fusarium oxysporum* species complex; ITS, Internal transcribed spacer; TEF1, elongation factor 1‐apha; RPB2, second largest subunit of RNA polymerase II. GenBank reference accession indicates the closest‐matching reference sequence retrieved from GenBank by BLAST. Sequence accession number indicates the GenBank accession assigned to the sequence generated in this study.
**Table S4:** Summary of in vitro antifungal susceptibility according to *Fusarium* species complex.
**Table S5:** Reference nucleotide sequences used for *CYP51A* primer design. Sequences were obtained from GenBank/RefSeq records or from publicly available genome annotations and were used to complement the multiple‐sequence alignments for the FSSC, FOSC and FFSC. FSSC, *F. solani* species complex; FFSC, *F. fujikuroi* species complex; FOSC, *F. oxysporum* species complex.
**Table S6:** Amino acid states in CYP51A of *Fusarium* clinical isolates at positions equivalent to *A. fumigatus* Cyp51A, with corresponding azole MIC values and GenBank accession numbers. FSSC, *Fusarium solani* species complex; FFSC, *Fusarium fujikuroi* species complex; FOSC, *Fusarium oxysporum* species complex; MIC, minimal inhibitory concentration; AMB (32–0.06 μg/mL), amphotericin B; VOR (32–0.06 μg/mL), voriconazole; ITR (32–0.06 μg/mL), itraconazole; POS (32–0.06 μg/mL), posaconazole; FLU (128–0.025 μg/mL), fluconazole.
**Table S7:** Fold‐change values of *CYP51A* and *ABC1* expression across biological replicates after voriconazole exposure (8 μg/mL for 6 h). For each biological replicate, expression is presented as mean fold‐change ± standard deviation (SD) calculated from technical triplicates. Relative expression was determined by the 2^−ΔΔCq^ method using untreated cultures as calibrators and GPD as the housekeeping gene. FSSC, *Fusarium solani* species complex; FOSC, *Fusarium oxysporum* species complex; SD, standard deviation.

## Data Availability

The data that support the findings of this study are available from the corresponding author upon reasonable request.
