# Supplementary material for: A Multifactorial Azole Response in Clinically Relevant Fusarium Species Complexes
Source: Mycoses. 2026 Sep 5;69(9):e70220. doi: 10.1111/myc.70220 (PMC13545660; doi:10.1111/myc.70220)
Supplement: Supplementary file 1 [file MYC-69-e70220-s001.docx]

**A multifactorial azole response in clinically relevant *Fusarium* species complexes**

Running Title: **Azole response in *Fusarium* species complexes**

Patrícia Helena Grizante Barião^1^, Ludmilla Tonani^1^, Adriana Rodrigues Alves^1^, Otávio Guilherme Gonçalves de Almeida^1^, Cledir Santos^2,3^, Marcia Regina von Zeska Kress^1*^

^1^ Department of Clinical Analyses, Toxicology, and Food Science, School of Pharmaceutical Sciences of Ribeirao Preto, University of Sao Paulo, Ribeirao Preto, SP, 14040-903, Brazil;

^2^ Scientific and Technological Bioresource Nucleus (BIORENUFRO), Universidad de La Frontera, Temuco 4811230, Chile

^3^ Postgraduate Program in Biotechnology, Federal University of Technology-Paraná, Ponta Grossa, PR, Brazil

*Corresponding author: [kress@fcfrp.usp.br](mailto:kress@fcfrp.usp.br)

**Supplementary material**

FOSC

FFSC

FSSC

**Figure S1. UPGMA tree of *CYP51A* reference sequences used for primer design across the FSSC, FOSC, and FFSC.** UPGMA tree inferred from *CYP51A* reference sequences used for primer design across the *Fusarium solani* species complex (FSSC), *Fusarium oxysporum* species complex (FOSC), and *Fusarium fujikuroi* species complex (FFSC). The tree shows clustering of the selected sequences according to species-complex level similarity, supporting the identification of conserved regions for subsequent alignment and primer design. Bootstrap values are shown at the nodes, and the scale bar represents genetic distance. Ffal, *F. falciforme*; Fke, *F. keratoplasticum*; Fpro, *F. proliferatum*; Ffu, *F. fujikuroi*; Fox, *F. oxysporum.*

**Table S1: Clinical isolates of *Fusarium* spp. and their biological sites of isolation.** FSSC, *F. solani* species complex; FFSC, *F. fujikuroi* species complex; FOSC, *F. oxysporum* species complex.

| **Species complex** | **Clinical Isolates** | **Species** | **Biological site** |
| --- | --- | --- | --- |
|  |  |  |  |
| FSSC | LMC7162.02 | *F. petroliphilum* | Blood culture |
|  | LMC7168.01 | *F. petroliphilum* | Blood culture |
|  | LMC7169.01 | *F. petroliphilum* | Blood culture |
|  | LMC7193.01 | *F. falciforme* | Great toenail |
|  | LMC7108.01 | *F. keratoplasticum* | Toenails |
|  | LMC7122.03 | *F. keratoplasticum* | Interdigital foot lesion |
|  | LMC7178.01 | *F. falciforme* | Great toenail |
|  | LMC7144.01 | *F. keratoplasticum* | Peritoneal fluid |
|  | LMC7148.01 | *F. keratoplasticum* | Peritoneal fluid |
|  | LMC7113.02 | *F. keratoplasticum* | Interdigital foot lesion |
|  | LMC7185.01 | *F. keratoplasticum* | Great toenail |
|  | LMC7188.01 | *F. keratoplasticum* | Great toenail |
|  | LMC7189.01 | *F. solani* | Great toenail |
|  | LMC7205.01 | *F. keratoplasticum* | Great toenail |
|  | LMC7210.01 | *F. suttonianum* | Skin lesion |
|  | LMC7214.01 | *F. keratoplasticum* | Great toenail |
|  | LMC7163.01 | *Fusarium* spp. | Blood culture |
| FFSC | LMC7138.01 | *F. proliferatum* | Great toenail |
|  | LMC21300.01 | *F. proliferatum* | Catheter-drawn blood |
|  | LMC21303.01 | *F. sacchari* | Catheter-drawn blood |
| FOSC | LMC7137.01 | *F. oxysporum* | Toenails |
|  | LMC7111.02 | *F. oxysporum* | Great toenail |
|  | LMC7117.01 | *F. oxysporum* | Fingernails |
|  | LMC7130.01 | *F. oxysporum* | Great toenail |
|  | LMC7172.01 | *F. oxysporum* | Toenails |
|  | LMC7170.01 | *F. oxysporum* | Blood culture |

**Table S2: Primer sets used for RT-qPCR and amplification efficiencies.** FSSC, *F. solani* species complex; FOSC, *F. oxysporum* species complex.

| **Primer pair** | **Target gene** | **Species complex** | **Concentration (ng)** | **gDNA Template** | **Efficiency (%)** |
| --- | --- | --- | --- | --- | --- |
| NkCYP51A-983-F | *CYP51A* | FSSC | 150 | *F. keratoplasticum* (LMC7205.01) | 103.98 |
| FSSCCYP51A-R |  |  | 150 |  |  |
| FOSCCYP51A-F | *CYP51A* | FOSC | 150 | *F. oxysporum* (LMC7117.01) | 103.09 |
| FOSCCYP51A-R |  |  | 150 |  |  |
| FkABC1-F3561 | *ABC1* | FSSC | 300 | *F. keratoplasticum* (LMC7205.01) | 105.35 |
| FkABC1-R3631 |  |  | 150 |  |  |
| NkGPD1-829-F | *GPD* | FSSC  and  FOSC | 300 | *F. keratoplasticum* (LMC7205.01) and *F. oxysporum* (LMC7117.01) | 104.44 |
| NkGPD1-1050-R |  |  | 150 |  |  |

**Table S3: BLAST-based molecular identification of *Fusarium* clinical isolates using ITS, *TEF1*, and *RPB2* sequences, including GenBank reference accessions and GenBank accession numbers of sequences generated in this study.** FSSC, *Fusarium solani* Species complex; FFSC, *Fusarium fujikuroi* Species complex; FOSC, *Fusarium oxysporum* Species complex; ITS, Internal transcribed spacer; TEF1, elongation factor 1-apha; RPB2, second largest subunit of RNA polymerase II. GenBank reference accession indicates the closest-matching reference sequence retrieved from GenBank by BLAST. Sequence accession number indicates the GenBank accession assigned to the sequence generated in this study.

| **Species complex** | **Isolates/strains** | **Species** | **ITS** | |  | |  | |  | ***TEF1*** | | |  | |  | |  | ***RPB2*** | | |  | |  | |  |
| --- | --- | --- | --- | --- | --- | --- | --- | --- | --- | --- | --- | --- | --- | --- | --- | --- | --- | --- | --- | --- | --- | --- | --- | --- | --- |
|  |  |  | **E-value** | **Identity (%)** | | **GenBank**  **reference accession** | | **GenBank**  **sequence**  **accession**  **number** | | | **E-value** | **Identity (%)** | | **GenBank**  **reference accession** | | **GenBank**  **sequence**  **accession**  **number** | | | **E-value** | **Identity (%)** | | **GenBank**  **reference accession** | | **GenBank**  **sequence**  **accession**  **number** | |
| FSSC | LMC7162.02 | *F. petroliphilum* | 0.0 | 100 | | MF467472 | | PV607006 | | | 0.0 | 100 | | MF467469 | | PV690776 | | | 0.0 | 100 | | MF467494 | | PV761219 | |
|  | LMC7168.01 | *F. petroliphilum* | 0.0 | 100 | | MF467472 | | PV607005 | | | 0.0 | 100 | | MF467469 | | PV690775 | | | 0.0 | 100 | | MF467494 | | PV761218 | |
|  | LMC7169.01 | *F. petroliphilum* | 0.0 | 99.77 | | MF467472 | | PV607007 | | | 0.0 | 100 | | MF467469 | | PV690777 | | | 0.0 | 100 | | MF467494 | | PV761220 | |
|  | LMC7193.01 | *F. falciforme* | 0.0 | 100 | | MF467480 | | PV607012 | | | 0.0 | 100 | | MF467462 | | PV690782 | | | 0.0 | 100 | | MF467484 | | PV761225 | |
|  | LMC7108.01 | *F. keratoplasticum* | 0.0 | 100 | | EF453072 | | PV607021 | | | 0.0 | 99.11 | | EF452920 | | PV690790 | | | 0.0 | 100 | | EF469959 | | PV761233 | |
|  | LMC7122.03 | *F. keratoplasticum* | 0.0 | 100 | | EF453072 | | PV607019 | | | 0.0 | 100 | | EF452920 | | PV690788 | | | 0.0 | 99.30 | | EF469959 | | PV761231 | |
|  | LMC7178.01 | *F. falciforme* | 0.0 | 100 | | MF467480 | | PV607015 | | | 0.0 | 99.10 | | MF467462 | | PV690785 | | | 0.0 | 100 | | MF467484 | | PV761228 | |
|  | LMC7144.01 | *F. keratoplasticum* | 0.0 | 100 | | EF453072 | | PV607018 | | | 0.0 | 98.21 | | EF452920 | | PV690787 | | | 0.0 | 99.30 | | EF469959 | | PV761230 | |
|  | LMC7148.01 | *F. keratoplasticum* | 0.0 | 100 | | EF453072 | | PV607017 | | | 0.0 | 98.21 | | EF452920 | | PV690786 | | | 0.0 | 99.30 | | EF469959 | | PV761229 | |
|  | LMC7113.02 | *F. keratoplasticum* | 0.0 | 100 | | EF453072 | | PV607020 | | | 0.0 | 99.11 | | EF452920 | | PV690789 | | | 0.0 | 99.30 | | EF469959 | | PV761232 | |
|  | LMC7185.01 | *F. keratoplasticum* | 0.0 | 100 | | EF453072 | | PV607014 | | | 0.0 | 99.85 | | EF452920 | | PV690784 | | | 0.0 | 99.30 | | EF469959 | | PV761227 | |
|  | LMC7188.01 | *F. keratoplasticum* | 0.0 | 99.57 | | EF453072 | | PV607008 | | | 0.0 | 100 | | EF452920 | | PV690778 | | | 0.0 | 99.30 | | EF469959 | | PV761221 | |
|  | LMC7189.01 | *F. solani* | 0.0 | 99.78 | | EF453097 | | PV607013 | | | 0.0 | 99.12 | | EF452945 | | PV690783 | | | 0.0 | 100 | | EF469984 | | PV761226 | |
|  | LMC7205.01 | *F. keratoplasticum* | 0.0 | 99.78 | | EF453072 | | PV607011 | | | 0.0 | 100 | | EF452920 | | PV690781 | | | 0.0 | 99.30 | | EF469959 | | PV761224 | |
|  | LMC7210.01 | *F. suttonianum* | 0.0 | 100 | | DQ094413 | | PV607010 | | | 0.0 | 98.95 | | DQ246944 | | PV690780 | | | 0.0 | 99.77 | | EU329574 | | PV761223 | |
|  | LMC7214.01 | *F. keratoplasticum* | 0.0 | 100 | | EF453072 | | PV607009 | | | 0.0 | 100 | | EF452920 | | PV690779 | | | 0.0 | 99.30 | | EF469959 | | PV761222 | |
|  | LMC7163.01 | *F. bostrycoides* | 0.0 | 99.82 | | OR538654 | | PZ441115 | | | 0.0 | 93.51 | | JF740784 | | PZ446721 | | | 0.0 | 99.89 | | MW684278 | | PZ446722 | |
|  | ATCC 36031 | *F. keratoplasticum* | 0.0 | 100 | | EF453072 | | PV607022 | | | 0.0 | 98.81 | | EF452920 | | PV690791 | | | 0.0 | 100 | | EF469959 | | PV761234 | |
| FFSC | LMC7138.01 | *F. proliferatum* | 0.0 | 100 | | U34558 | | PV607030 | | | 0.0 | 98.76 | | AF160280 | | PV690799 | | | 0.0 | 100 | | JX171617 | | PV761242 | |
|  | LMC21300.01 | *F. proliferatum* | 0.0 | 100 | | U34558 | | PV607031 | | | 0.0 | 98.61 | | AF160280 | | PV690800 | | | 0.0 | 99.67 | | JX171617 | | PV761243 | |
|  | LMC21303.01 | *F. sacchari* | 0.0 | 100 | | KU604026 | | PV607032 | | | 0.0 | 99.39 | | MW402031 | | PV690801 | | | 0.0 | 99.15 | | MW402793 | | PV761244 | |
| FOSC | LMC7137.01 | *F. oxysporum* | 0.0 | 100 | | EU721709 | | PV607023 | | | 0.0 | 98.87 | | MH485045 | | PV690792 | | | 0.0 | 99.66 | | MH484954 | | PV761235 | |
|  | LMC7111.02 | *F. oxysporum* | 0.0 | 100 | | EU721709 | | PV607024 | | | 0.0 | 98.70 | | MH485045 | | PV690793 | | | 0.0 | 99.66 | | MH484954 | | PV761236 | |
|  | LMC7117.01 | *F. oxysporum* | 0.0 | 100 | | EU721709 | | PV607025 | | | 0.0 | 98.54 | | MH485045 | | PV690794 | | | 0.0 | 99.66 | | MH484954 | | PV761237 | |
|  | LMC7130.01 | *F. oxysporum* | 0.0 | 99.80 | | EU721709 | | PV607026 | | | 0.0 | 99.03 | | MH485045 | | PV690795 | | | 0.0 | 99.09 | | MH484954 | | PV761238 | |
|  | LMC7172.01 | *F. oxysporum* | 0.0 | 100 | | EU721709 | | PV607027 | | | 0.0 | 97.40 | | MH485045 | | PV690796 | | | 0.0 | 99.54 | | MH484954 | | PV761239 | |
|  | LMC7170.01 | *F. oxysporum* | 0.0 | 100 | | EU721709 | | PV607028 | | | 0.0 | 99.03 | | MH485045 | | PV690797 | | | 0.0 | 99.54 | | MH484954 | | PV761240 | |
|  | ATCC 48112 | *F. oxysporum* | 0.0 | 99.80 | | EU721709 | | PV607029 | | | 0.0 | 98.87 | | MH485045 | | PV690798 | | | 0.0 | 99.66 | | MH484954 | | PV761241 | |

**Table S4: Summary of *in vitro* antifungal susceptibility according to *Fusarium* species complex.** Minimum inhibitory concentration (MIC) data for amphotericin B (AMB), voriconazole (VOR), itraconazole (ITR), and posaconazole (POS) according to *Fusarium* species complex. AFG, antifungal; FSSC, *Fusarium solani* species complex; FOSC, *Fusarium oxysporum* species complex; FFSC, *Fusarium fujikuroi* species complex; MIC50 and MIC90 represent the lowest concentrations inhibiting 50% and 90% of the isolates, respectively. GM, geometric mean; ND, not determined because of the low number of isolates; NC, not calculated due to off-scale MIC values (>32 µg/mL) and limited interpretability of the geometric mean under these conditions.

| **AFG** | **Parameter**  (µg/mL) | **FSSC** | **FOSC** | **FFSC** |
| --- | --- | --- | --- | --- |
|  |  | (n = 17) | (n = 6) | (n = 3) |
| AMB | MIC Range | 1.0 to >32 | 2.0 | 2.0 |
|  | MIC50 | 2.0 | 2.0 | ND |
|  | MIC90 | 2.0 | 2.0 | ND |
|  | GM | 1.8 | 2.0 | 2.0 |
| VOR | MIC Range | 2.0 to >32 | 2.0 to 8.0 | 4.0 to 8.0 |
|  | MIC50 | 32 | 4.0 | ND |
|  | MIC90 | >32 | 4.0 | ND |
|  | GM | 8.0 | 3.6 | 5.0 |
| ITR | MIC Range | >32 | >32 | >32 |
|  | MIC50 | >32 | >32 | ND |
|  | MIC90 | >32 | >32 | ND |
|  | GM | NC | NC | NC |
| POS | MIC Range | >32 | 2.0 to >32 | 2.0 to >32 |
|  | MIC50 | >32 | >32 | ND |
|  | MIC90 | >32 | >32 | ND |
|  | GM | NC | 2.8 | NC |

**Table S5: Reference nucleotide sequences used for *CYP51A* primer design.** Sequences were obtained from GenBank/RefSeq records or from publicly available genome annotations and were used to complement the multiple-sequence alignments for the FSSC, FOSC, and FFSC. FSSC, *F. solani* species complex; FFSC, *F. fujikuroi* species complex; FOSC, *F. oxysporum* species complex.

| Species complex | Species | Accession / identifier | Source |
| --- | --- | --- | --- |
| FSSC | *F. falciforme* | MN640610.1 | GenBank |
|  | *F. keratoplasticum* | MN296717.1 | GenBank |
|  | *F. keratoplasticum* | MN296719.1 | GenBank |
|  | *F. keratoplasticum* | MN296714.1 | GenBank |
|  | *F. keratoplasticum* | MN296713.1 | GenBank |
|  | *F. keratoplasticum* | MN296712.1 | GenBank |
| FFSC | *F. proliferatum* | XM_031222743.1 | GenBank/RefSeq |
|  | *F. fujikuroi* | XM_023568553.1 | GenBank/RefSeq |
| FOSC | *F. oxysporum f. sp.* *lycopersici* | XM_018391222.1 | GenBank/RefSeq |
|  | *F. oxysporum* | XM_031190304.1 | GenBank/RefSeq |
|  | *F. oxysporum* | 45GCA_900096695.1 | Public genome annotation |
|  | *F. oxysporum* | FMJY01000008.1 | Public Genome sequence |
|  | *F. oxysporum* | Chr13.g2683.t1 | Public genome annotation |
|  | *F. oxysporum* f. sp. *vasinfectum* | JH657929.1 | GenBank |

**Table S6: Amino acid states in CYP51A of *Fusarium* clinical isolates at positions equivalent to *A. fumigatus* Cyp51A, with corresponding azole MIC values and GenBank accession numbers.** FSSC, *Fusarium solani* species complex; FFSC, *Fusarium fujikuroi* species complex; FOSC, *Fusarium oxysporum* species complex; MIC, minimal inhibitory concentration; AMB (32 – 0.06 µg/mL), amphotericin B; VOR (32 – 0.06 µg/mL), voriconazole; ITR (32 – 0.06 µg/mL), itraconazole; POS (32 – 0.06 µg/mL), posaconazole; FLU (128 – 0.025 µg/mL), fluconazole.

| **Species complex** | **Isolate/strain** | **Species** | **CYP51A amino acid states** | | | | | | | **MIC (µg/mL)** | | | | **GenBank sequence accession number** |
| --- | --- | --- | --- | --- | --- | --- | --- | --- | --- | --- | --- | --- | --- | --- |
|  |  |  | **52** | **88** | **98** | **172** | **220** | **255** | **427** | **ITR** | **VOR** | **POS** | **FLU** |  |
| FSSC | LMC7162.02 | *F. petroliphilum* | S | E | L | V | L | E | D | >32 | 32 | >32 | >128 | PV761247 |
|  | LMC7168.01 | *F. petroliphilum* | S | E | L | V | L | E | D | >32 | 4 | >32 | >128 | PV761246 |
|  | LMC7169.01 | *F. petroliphilum* | S | E | L | V | L | E | D | >32 | >32 | >32 | >128 | PV761248 |
|  | LMC7193.01 | *F. falciforme* | S | E | L | A | L | E | D | >32 | 32 | >32 | >128 | PV761253 |
|  | LMC7108.01 | *F. keratoplasticum* | S | E | L | V | L | E | D | >32 | 8 | >32 | >128 | PV761261 |
|  | LMC7122.03 | *F. keratoplasticum* | S | E | L | V | L | E | D | >32 | >32 | >32 | >128 | PV761259 |
|  | LMC7178.01 | *F. falciforme* | S | E | L | V | L | E | D | >32 | 16 | >32 | >128 | PV761256 |
|  | LMC7144.01 | *F. keratoplasticum* | S | E | L | V | L | E | D | >32 | 2 | >32 | >128 | PV761258 |
|  | LMC7148.01 | *F. keratoplasticum* | S | E | L | V | L | E | D | >32 | 2 | >32 | >128 | PV761257 |
|  | LMC7113.02 | *F. keratoplasticum* | S | E | L | V | L | E | D | >32 | >32 | >32 | >128 | PV761260 |
|  | LMC7185.01 | *F. keratoplasticum* | S | E | L | V | L | E | D | >32 | 8 | >32 | >128 | PV761255 |
|  | LMC7188.01 | *F. keratoplasticum* | S | E | L | V | L | E | D | >32 | 8 | >32 | >128 | PV761249 |
|  | LMC7189.01 | *F. solani* | S | E | L | V | L | E | D | >32 | 2 | >32 | >128 | PV761254 |
|  | LMC7205.01 | *F. keratoplasticum* | S | E | L | V | L | E | D | >32 | >32 | >32 | >128 | PV761252 |
|  | LMC7210.01 | *F. suttonianum* | S | E | L | V | L | E | D | >32 | >32 | >32 | >128 | PV761251 |
|  | LMC7214.01 | *F. keratoplasticum* | S | E | L | V | L | E | D | >32 | >32 | >32 | >128 | PV761250 |
|  | LMC7163.01 | *F. bostrycoides* | S | E | L | V | L | E | D | >32 | 32 | >32 | >128 | PZ535933 |
|  | ATCC 36031 | *F. keratoplasticum* | S | E | L | V | L | E | D | >32 | 16 | >32 | >128 | PV761262 |
| FFSC | LMC7138.01 | *F. proliferatum* | A | D | L | V | L | G | E | >32 | 4 | 2 | >128 | PV761270 |
|  | LMC21300.01 | *F. proliferatum* | A | D | L | V | L | G | E | >32 | 8 | >32 | >128 | PV761271 |
|  | LMC21303.01 | *F. sacchari* | A | D | I | V | L | G | E | >32 | 4 | >32 | >128 | PV761272 |
| FOSC | LMC7137.01 | *F. oxysporum* | A | D | I | V | L | G | D | >32 | 4 | 2 | >128 | PV761263 |
|  | LMC7111.02 | *F. oxysporum* | A | D | I | V | L | G | D | >32 | 2 | >32 | >128 | PV761264 |
|  | LMC7117.01 | *F. oxysporum* | A | D | I | V | L | G | D | >32 | 4 | >32 | >128 | PV761265 |
|  | LMC7130.01 | *F. oxysporum* | A | D | I | V | L | G | D | >32 | 8 | >32 | >128 | PV761266 |
|  | LMC7172.01 | *F. oxysporum* | A | D | I | V | L | G | D | >32 | 4 | >32 | >128 | PV761267 |
|  | LMC7170.01 | *F. oxysporum* | A | D | I | V | L | G | D | >32 | 2 | 4 | >128 | PV761268 |
|  | ATCC 48112 | *F. oxysporum* | A | D | I | V | L | G | D | >32 | 16 | >32 | >128 | PV761269 |

**Table S7. Fold-change values of *CYP51A* and *ABC1* expression across biological replicates after voriconazole exposure (8 µg/mL for 6 h).** For each biological replicate, expression is presented as mean fold-change ± standard deviation (SD) calculated from technical triplicates. Relative expression was determined by the 2^-ΔΔCq^ method using untreated cultures as calibrators and GPD as the housekeeping gene. FSSC, *Fusarium solani* species complex; FOSC, *Fusarium oxysporum* species complex; SD, standard deviation.

| **Species complex** | **Isolate/strain** | **Species** | **Gene** | **Biological replicate 1 (mean ± SD)** | **Biological replicate 2 (mean ± SD)** | **Biological replicate 3 (mean ± SD)** |
| --- | --- | --- | --- | --- | --- | --- |
| FSSC | ATCC 36031 | *F. keratoplasticum* | *CYP51A* | 168.24 ± 3.15 | 13.42 ± 0.45 | 16.85 ± 0.69 |
| FSSC | LMC7108.01 | *F. keratoplasticum* | *CYP51A* | 13.88 ± 0.53 | 7.30 ± 0.57 | 6.40 ± 0.86 |
| FSSC | LMC7113.02 | *F. keratoplasticum* | *CYP51A* | 483.52 ± 3.28 | 68.14 ± 2.69 | 22.74 ± 1.73 |
| FOSC | ATCC 48112 | *F. oxysporum* | *CYP51A* | 3785.37 ± 17.37 | 458.76 ± 40.52 | 200.24 ± 12.05 |
| FOSC | LMC7137.01 | *F. oxysporum* | *CYP51A* | 6443.7 ± 44.45 | 584.61 ± 16.32 | 464.01 ± 17.60 |
| FSSC | ATCC 36031 | *F. keratoplasticum* | *ABC1* | 2.49 ± 0.24 | 3.07 ± 0.7 | 1.89 ± 0.29 |
| FSSC | LMC7108.01 | *F. keratoplasticum* | *ABC1* | 11.21 ± 0.98 | 32.23 ± 1.56 | 10.23 ± 1.03 |
| FSSC | LMC7113.02 | *F. keratoplasticum* | *ABC1* | 6.91 ± 0.34 | 4.85 ± 0.67 | 3.58 ± 0.14 |
